# On Connected Target *k*-Coverage in Heterogeneous Wireless Sensor Networks

**DOI:** 10.3390/s16010104

**Published:** 2016-01-15

**Authors:** Jiguo Yu, Ying Chen, Liran Ma, Baogui Huang, Xiuzhen Cheng

**Affiliations:** 1 School of Information Science and Engineering, Qufu Normal University, Rizhao 276826, Shandong, China; Ying_Chen2014@126.com (Y.C.); hjbaogui@163.com (B.H.); 2 Department of Computer Science, Texas Christian University, Fort Worth, TX 298850, USA; l.ma@tcu.edu; 3 Department of Computer Science, The George Washington University, Washington, DC 20052, USA; cheng@gwu.edu

**Keywords:** heterogeneous wireless sensor networks (HWSNs), *k*-coverage, connectivity, connected cover set

## Abstract

Coverage and connectivity are two important performance evaluation indices for wireless sensor networks (WSNs). In this paper, we focus on the connected target *k*-coverage (CTC${}_{k}$) problem in heterogeneous wireless sensor networks (HWSNs). A centralized connected target *k*-coverage algorithm (CCTC${}_{k}$) and a distributed connected target *k*-coverage algorithm (DCTC${}_{k}$) are proposed so as to generate connected cover sets for energy-efficient connectivity and coverage maintenance. To be specific, our proposed algorithms aim at achieving minimum connected target *k*-coverage, where each target in the monitored region is covered by at least *k* active sensor nodes. In addition, these two algorithms strive to minimize the total number of active sensor nodes and guarantee that each sensor node is connected to a sink, such that the sensed data can be forwarded to the sink. Our theoretical analysis and simulation results show that our proposed algorithms outperform a state-of-art connected *k*-coverage protocol for HWSNs.

## 1. Introduction

A wireless sensor network (WSN) is an auto-configured network consisting of a large number of sensors deployed in a monitored area in a random or deterministic manner. WSNs are used extensively in various scenarios, such as military applications [1], environmental monitoring [2], target surveillance [3] and disaster prevention [4]. The sensors in WSNs can sense and collect raw data from the environment, perform local processing, possibly communicate with each other, so as to perform aggregation [5], and route the aggregated data to sinks.

In a heterogeneous wireless sensor network (HWSN), sensor nodes may have different capabilities in terms of transmit power, sensing capabilities and battery life. Most of the existing works on coverage and connectivity assume that the sensing range and the communication range follow a binary disk model. However, empirical observations have shown that this model is far from reality [6,7]. In this paper, we consider a more realistic model for the sensing and communication ranges of nodes. More precisely, for the tractability of the problem, we consider a circular model with different sensing and communication radii and an irregular convex model with different kinds of sensing and communication ranges.

For a WSN, coverage and connectivity are two key factors. Target coverage is to monitor a set of targets with a subset of the available sensor nodes. A node failure may render the entire network disconnected. Applications requiring *k*-coverage may occur in situations (e.g., military applications) where a stronger environmental monitoring capability is desired. Such a problem can be formulated as a decision problem, whose goal is to determine whether each target in the target set (or each point in the sensing region) is covered by at least *k* sensors, where *k* is a predefined value. The problem of target *k*-coverage is to guarantee the network reliability and accuracy. Along with coverage, the notion of network connectivity is equally fundamental to a sensor network design. The sensor nodes cannot deliver the sensed data to the sinks without network connectivity. The connected target *k*-coverage (CTC${}_{k}$) problem takes *k*-coverage and connectivity into consideration simultaneously, which is one of the target coverage (TC) problems.

Due to the limited energy of sensor nodes and the difficulty of replacing or recharging their batteries, it is necessary for the sensor nodes to be densely deployed. However, keeping all of the nodes on all of the time could deplete their energy quickly. Hence, we adopt the duty-cycled mode in this work, so as to save energy and extend the network lifetime. To be specific, the nodes are divided into a number of subsets, called cover sets, where each cover set is capable of covering all of the monitored targets. Cover sets can be classified into disjoint cover sets and non-disjoint cover sets. It has been shown that the use of non-disjoint cover sets may increase the lifetime of the network, if proper scheduling algorithms are used [8].

**Figure 1:**
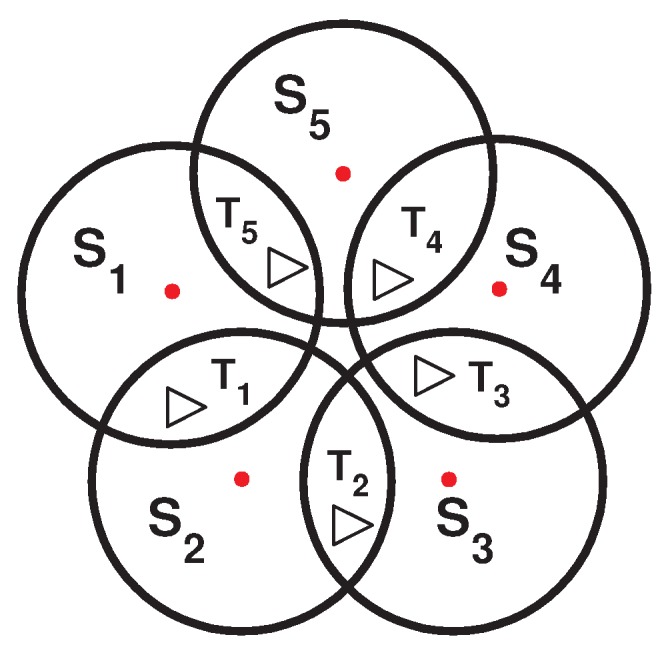
Example topology for sensors and targets.

As shown in Figure 1, we assume that the five targets (T${}_{1}$, T${}_{2}$, T${}_{3}$, T${}_{4}$ and T${}_{5}$) lie on a field that can be monitored by five sensor nodes (S${}_{1}$, S${}_{2}$, S${}_{3}$, S${}_{4}$ and S${}_{5}$). Node S${}_{1}$ covers T${}_{1}$ and T${}_{5}$; node S${}_{2}$ covers T${}_{1}$ and T${}_{2}$, node S${}_{3}$ covers T${}_{2}$ and T${}_{3}$, node S${}_{4}$ covers T${}_{3}$ and T${}_{4}$; and node S${}_{5}$ covers T${}_{4}$ and T${}_{5}$. If all of the sensors in a terrain are activated at the same time, the lifetime of the network would be equal to the lifetime of a single sensor (assuming that all of the sensors have identical energy resources), say h hours.

**Figure 2:**
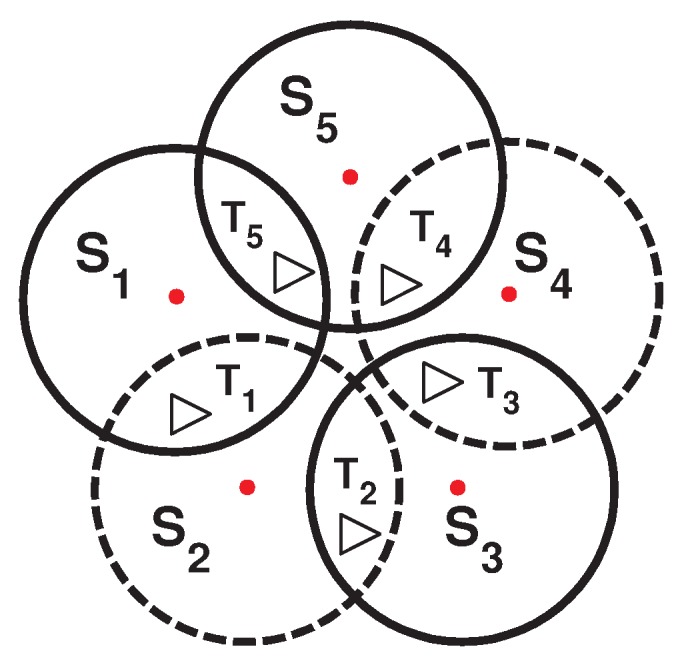
One disjoint cover set generated.

**Figure 3:**
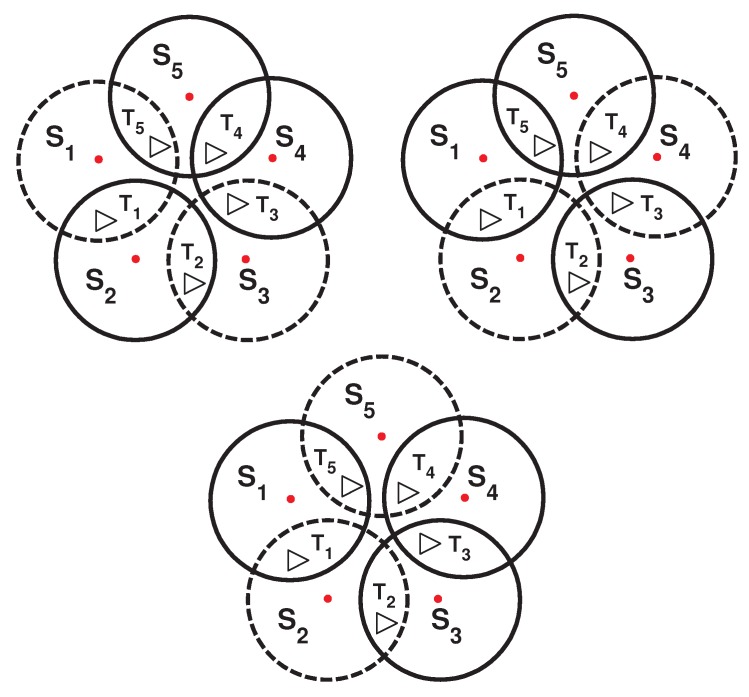
Three non-disjoint cover sets generated.

As shown in Figure 2, by dividing the sensors into disjoint sets, the resulting network lifetime would still be h, since for this topology, a disjoint algorithm can produce only one cover set (S${}_{1}$, S${}_{3}$, S${}_{5}$). However, if a sensor node in Figure 1 can be part of two cover sets, the network lifetime can be extended. By creating three non-disjoint cover sets (see Figure 3), each one activated for 0.5 h, the total network lifetime can be extended to 1.5 h, assuming that the energy consumption during sleep mode is negligible.

Traditionally, a centralized algorithm requires each sensor node to forward all of its observed data to the sinks, resulting in large energy consumption in communication. A distributed algorithm, on the other hand, allows each sensor node to decide its own working mode according to the information that has been gathered by its neighbors. Compared to centralized algorithms, distributed algorithms can reduce communication energy, enhance detection accuracy and increase the processing capacity. As a result, we adopt the distributed algorithms in this paper, as it is more suitable for large-scale networks.

The main contributions of this work can be summarized as follows. For energy-efficient target *k*-coverage and connectivity of HWSNs, we propose a centralized connected target *k*-coverage algorithm (CCTC${}_{k}$) and a distributed connected target *k*-coverage algorithm (DCTC${}_{k}$). Different from the existing work, CCTC${}_{k}$ and DCTC${}_{k}$ have the following advantages.

1. An irregular convex sensing and communication model is proposed.
2. CCTC${}_{k}$ and DCTC${}_{k}$ maintain target *k*-coverage, so as to guarantee network reliability and accuracy.
3. CCTC${}_{k}$ and DCTC${}_{k}$ can achieve the load balance of sensor nodes and prolong the network lifetime by considering the sensing capabilities, remaining battery life and the *k*-coverage targets of each sensor node.
4. DCTC${}_{k}$ is distributed so that it an be easily implemented in large-scale HWSNs.

The rest of the paper is organized as follows. Section 2 introduces the related works about target coverage. We lay out the problem description in Section 3 and describe our proposed centralized connected target *k*-coverage algorithm (CCTC${}_{k}$) and distributed connected target *k*-coverage algorithm (DCTC${}_{k}$) in Section 4. We analyze the performance of CCTC${}_{k}$ and DCTC${}_{k}$ in Section 5 and present the simulation results in Section 6. Finally, we conclude the paper in Section 7.

## 2. Related Work

Coverage and connectivity are two fundamental problems in wireless sensor networks. The coverage problem either deploys sensors to cover the desired area, or selects active sensors and schedules them to cover the desired area, or analyzes how well the desired area is covered; while the connectivity problem is that each node can communicate with sinks either directly or indirectly through other nodes, such that the sensed data can be forwarded to them. Recently, the coverage and connectivity problems have received extensive attention. In this section, we present a brief overview of the related work on coverage and connectivity in wireless sensor networks.

Coverage and connectivity have been extensively studied in [9,10,11,12]. Yet, little attention has been paid to that of HWSNs. In [13], Wang *et al.* proposed a fine analysis of coverage using two types of nodes with different capabilities and discussed the impact of heterogeneous sensing and communication ranges of the nodes on coverage and broadcast reachability. In [14], Lazos *et al.* addressed the problem of stochastic coverage in heterogeneous sensor networks. To derive analytical expressions of coverage for heterogeneous sensor networks, they formulated the coverage problem as a set intersection problem. In [15], Lin *et al.* considered the *k*-coverage problem in hybrid sensor networks, where each node is equipped with various types of sensors. They found that the optimal solution for the *k*-coverage was a pure Nash equilibrium, and thus, an algorithm was designed based on game theory. In [16], Du *et al.* proposed a differentiated coverage algorithm to overcome the poor scalability and performance bottleneck of HWSNs, where different network areas are provided with different degrees of sensing coverage. The motivation is that different network areas do not have the same importance, and hence, some areas require a higher coverage degree than others.

In [17], Zhao and Gurusamy modeled the connected target coverage as a maximum cover tree (MCT) problem. They showed the NP-completeness of the MCT problem and provided an upper bound lifetime for the MCT problem. They also proposed a greedy heuristic communication weighted greedy cover that runs in three phases to guarantee coverage and connectivity. In [18], Zorbas and Douligeris addressed the connected target coverage problem. They provided a generic greedy heuristic algorithm to solve the problem. In [19], Yu *et al.* studied the *k*-coverage scheduling problem to guarantee *k*-coverage sensing and network connectivity under both deterministic and stochastic sensing models of the sensors. In [20], Liu *et al.* addressed the problem of achieving energy conservation, coverage and connectivity requirements together in WSNs for vehicular applications. They proved such a problem is NP-complete and presented two algorithms to generate maximum disjoint sets. In [21], Yu *et al.* proposed a connected *k*-coverage working set construction algorithm (CWSC), which can produce different coverage degrees according to different applications, and, thus, can enhance the flexibility of the sensor network.

In distributed coverage algorithms, a number of sensor nodes perform the required calculations cooperatively and then disseminate the scheduling information to other sensor nodes. In [22], Gallais *et al.* used a distributed and localized scheduling scheme to rapidly disseminate the scheduling information throughout the network. The proposed scheme maintains connected area coverage under various ratios of communicating and sensing radii. There are extra costs associated with this kind of algorithm, due to the increased overhead in message exchanges and the need for the synchronization of participating nodes. In [23], Shih *et al.* considered the connected target coverage problem with multiple sensing units, which could be reduced to a connected set cover problem and further formulated as an integer linear programming (ILP) problem. They proposed two distributed heuristic schemes, the remaining energy first scheme (REFS) and the energy efficiency first scheme (EEFS).

Recently, target coverage problems have attracted extensive attention. In [24], such a problem with multiple sensing units was investigated, which is similar to the work in [23]. Yet, the connectivity issue was not taken into account. In [25], Yang *et al.* formalized the *k*-connected coverage set problems, developed a linear programming algorithm and designed two non-global solutions for them, cluster-based and pruning-based. In [26], the connected target coverage problem was considered, where the network consists of two types of sensors. One is the resource-rich sensors called supernodes used for data relaying, and the other is the energy-constrained sensors. In the paper, supernodes are assumed to have two transceivers, one for communication with sensors and the other for communication with other supernodes. All supernodes form a connected network, and each active sensor node connects to at least one supernode. A centralized algorithm, as well as a distributed algorithm are proposed in the paper.

Due to the limited lifetime of sensors, it may not be feasible to recharge or replace sensors in many situations. Thus, the network lifetime is also an important topic in WSNs. In [27], Zorbas *et al.* proposed a centralized algorithm for the target coverage problem to produce both disjoint cover sets and non-disjoint cover sets in homogeneous WSNs. In [28], Mostafaei and Meybodi proposed an efficient scheduling method based on learning automata, where each node is equipped with a learning automaton, which helps the node to select its proper state (active or sleep) at any given time. In [29], Diop *et al.* proposed a sensor-scheduling mechanism in probabilistic target coverage. The target detection model is based on the path loss lognormal shadowing model [30], which takes into account the distance parameter and the sensor’s physical characteristics. The major change in this study compared to earlier works lies in the consideration of a non-idealistic sensor coverage model. They attempt to partition sensors into a maximum number of set covers that guarantee full targets coverage. In [31], Deng *et al.* discussed the energy-efficient area coverage problem considering boundary effects in a new perspective.

Coverage and connectivity have been extensively studied, especially the network lifetime. While most studies either consider the homogeneous wireless sensor networks or produce a disjoint cover set to prolong the network lifetime, Mostafaei and Meybodi proposed an efficient scheduling method based on learning automata, which can produce disjoint cover sets. Shih *et al*. proposed two distributed heuristic schemes, REFS and EEFS . The advantages of REFS are its simplicity and reduced communication overhead. Moreover, EEFS is proposed to utilize sensors’ energy efficiently. However, EEFS and REFS just consider sensor’s remaining energy. Unlike the aforementioned studies, we propose a novel centralized coverage and connectivity algorithm (CCTC${}_{k}$) and a distributed connected target *k*-coverage algorithm (DCTC${}_{k}$), which can produce both disjoint and non-disjoint connected cover sets. Both the CCTC${}_{k}$ and DCTC${}_{k}$ algorithms can achieve load balance of sensor nodes and prolong the network lifetime by considering the sensing capabilities, remaining battery life and the *k*-coverage targets of each sensor node. Simulation results show that our schemes can prolong the network lifetime and save energy effectively.

## 3. Problem Description

We assume that all of the sensor nodes and targets are randomly and uniformly deployed in a region. Sensors are static after deployment. These sensor nodes can achieve *k*-coverage for each target assuming the network formed by these sensor nodes is connected. Sensor nodes do not know their locations. The sensing range and the communication range of different sensor nodes are different. We also assume that certain sinks in the monitored region can have two radio transceivers. One of the transceivers is for communicating with sensor nodes and the other is for communicating with other sinks. Sinks are more expensive and, thus, have more power, higher data rates and stronger processing power, as well as larger storage capabilities than those of sensor nodes. The main task performed by a sink is to relay data from sensor nodes or other sinks to the servers outside the monitored region.

Definition 1: *k*-coverage. Given a sensor node set *S* and a target set *T*, if each target in *T* is covered by at least *k* sensor nodes in *S*, the target set *T* is *k*-covered by *S*.

Definition 2: connected cover set. The set composed of sensor nodes in the active state, satisfying both target coverage and network connectivity, is called a connected cover set. If each sensor node belongs to only one connected cover set, e.g., C${}_{i}$⋂ C${}_{j}=\emptyset$, these cover sets are disjoint connected cover sets. If some sensor nodes belong to different connected cover sets, e.g., C${}_{i}$⋂ C${}_{j}\neq \emptyset$, these cover sets are called non-disjoint connected cover sets.

The size of connected cover sets is an important measurement of the performance of a scheduling algorithm. The smaller a connected cover set is, the less the energy it consumes.

Definition 3: network lifetime. Network lifetime is the time interval from the activation of the network until a coverage hole appears or from the point that the network starts operation until the set of all of the sensor nodes with nonzero remaining energy is not a connected cover set.

Definition 4: homogeneous and heterogeneous WSNs. A WSN is homogeneous if all of its nodes have the same sensing range, the same communication range and the same initial energy. Otherwise, it is heterogeneous. 

WSNs involve two different issues, connectivity and coverage. Each sensor node is equipped with at least one sensing module, one computation module and one communication module. These modules enable the collection of monitoring data and the delivery of these data to sinks. Connectivity is determined by the nodes’ on/off state of its communication modules, and coverage is determined by the nodes’ on/off state of sensing modules. We assume that each sensor can separately control the states of communication and sensing modules, *i.e.*, the state of the communication module is independent of the sensing module. As a result, there are five possible sensor states: (1) ready: the computation module is on-duty, and the sensing module and communication module are off-duty; (2) wait: the sensing module is off-duty, and the computation module and communication module are on-duty; (3) active: both the sensing module and communication module are on-duty. A sensor node can monitor the targets and communicate with other sensor nodes (or with sinks). Obviously, a sensor consumes much less energy in the sleep state than in the active state; (4) Sleep: the sensing module, computation module and communication module are all off-duty. In such a mode, a sensor node cannot monitor or transmit data; (5) Relay: the sensing module and computation module are off-duty, while the communication module is on-duty. The sensor nodes in such a mode are used to relay the sensed data.

**Figure 4:**
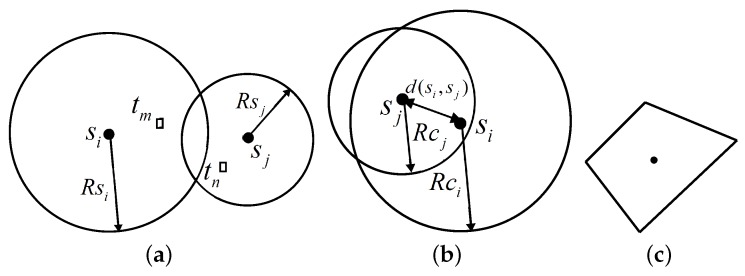
Sensing and communication models. **a**) Sensing model; (**b**) communication model; (**c**) convex sensing and communication model.

The simplest sensing model is the binary disk model, where a node is capable of sensing the targets that lie within its sensing range. Thus, in this model, the sensing range for the sensor node $S_{i}$ is confined within a circular disk of radius $Rs_{i}$ and $Rs_{i}$ is commonly called the sensing radius of the sensor node $S_{i}$. If the distance between a target $t_{i}$ and $S_{i}$ is less than $Rs_{i}$, *i.e.*, $d\left(S_{i},t_{i}\right)\leq Rs_{i}$, the target $t_{i}$ is covered by $S_{i}$. As shown in Figure 4a, the target $t_{m}$ is covered by the sensor node $S_{i}$, and the target $t_{n}$ is not covered by $S_{i}$, but covered by the node $S_{j}$.

The simplest communication model is also the binary disk model. It assumes that each node $S_{i}$ is able to communicate only up to a certain threshold distance from itself, called the communication radius, denoted by $Rc_{i}$, as illustrated in Figure 4b. Nodes can have different communication ranges depending on their transmission power. Two nodes $S_{i}$ and $S_{j}$ are able to communicate with each other if the Euclidean distance between them is less than or equal to the minimum of their communication radii, *i.e.*, $d\left(S_{i},S_{j}\right)\leq min\left\{Rc_{i},Rc_{j}\right\}$. Two such nodes that are able to communicate with each other are called one-hop neighbors and are said to have a communication edge between them. This is also known as the graph-based communication model.

The binary disk sensing and communication models can be extended to more realistic ones, the convex sensing and communication modules, as shown in Figure 4c.

To account for the practical characteristics of sensor nodes, we consider a more realistic model for the sensing and communication ranges. More precisely, for the tractability of the problem, we consider an arbitrary convex model, where both the sensing range and the communication range of sensor nodes are convex, but not necessarily circular. We adopt the notion of the largest enclosed disk and smallest enclosing disk [32]. 

Definition 5: The largest enclosed disk of the sensing range of a node $S_{i}$, say $A_{i}$, is a disk that lies inside $A_{i}$. The disk’s diameter is equal to the length of the shortest line segment among all lines segments passing through the location $p_{i}$ of $S_{i}$ and connecting between any pair of points on $A_{i}$’s boundary.

Definition 6: The smallest enclosing disk of $A_{i}$ is a disk whose diameter is equal to the length of the longest line segment among all possible line segments passing through the location $p_{i}$ of $S_{i}$ and connecting between any pair of points on $A_{i}$’s boundary.

**Figure 5:**
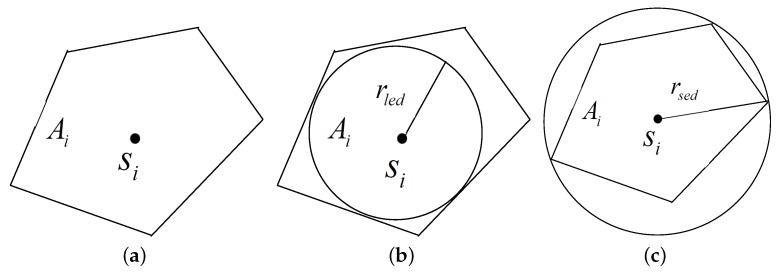
Convex sensing region. (**a**) Convex sensing range; (**b**) largest enclosed disk; (**c**) smallest enclosing disk.

Let $R_{led}$($S_{i}$) and $R_{sed}$($S_{i}$) be the radii of the largest enclosed disk and the smallest enclosing disk of the sensing range of node $S_{i}$, respectively, as shown in Figure 5. It is clear that any point in the largest enclosed disk of $S_{i}$ can be sensed by $S_{i}$. In this case, the coverage portion of CCTC${}_{k}$ and DCTC${}_{k}$ should use $R_{led}$ instead of Rs. Note that any other processing remains the same. Obviously, some targets would have a higher coverage degree than *k*. The main question is how to compute the value of $R_{led}$($S_{i}$) for a given node $S_{i}$. Intuitively, $R_{sed}$($S_{i}$) represents the distance between the farthest point that can be sensed by $S_{i}$ and the location $p_{i}$ of the node $S_{i}$. However, there is no guarantee that $S_{i}$ is able to sense all of the targets located within distance $R_{sed}$($S_{i}$) from $p_{i}$. It is clear that the performance of the coverage algorithm can be impacted by the choice of the radii of the sensing ranges of the nodes. We assume that these two attributes, *i.e.*, $R_{led}$($S_{i}$) and $R_{sed}$($S_{i}$), are computed based on experiments and are associated with each node $S_{i}$. In other words, $R_{led}$($S_{i}$) and $R_{sed}$($S_{i}$) are two of the characteristics that describe the behavior of the node $S_{i}$.

The convex communication model is similar to the convex sensing model.

We formulated this problem as the minimum connected target *k*-coverage (MCTC${}_{k}$). Given a target set *T*, a heterogeneous sensor set *S*, a set of sinks *B* and an integer k≥1, the goal is to select a minimum subset of heterogeneous sensor nodes of *S*, such that each target in *T* is *k*-covered, while all active nodes are connected to the sinks in *B*.

## 4. Algorithm Description

Due to the limited battery life of sensor nodes, it is necessary to design an energy efficient algorithm that can simultaneously reduce computation and communication overhead, provide a high degree of coverage and maintain a globally-connected network. To address this challenge, we propose two algorithms, CCTC${}_{k}$ and DCTC${}_{k}$, that can satisfy these conditions simultaneously for HWSNs with numerous sensor nodes and limited network lifetime. These algorithms are suitable for full target *k*-coverage problems, that is they can produce connected cover sets capable of monitoring all targets and to ensure that each target is monitored by at least *k* sensor nodes (k≥1).

Symbols and parameters used for CCTC${}_{k}$ and DCTC${}_{k}$ are summarized as follows. *S* is the set of sensor nodes deployed, and *n* is the total number of sensor nodes, *S*={$s_{1}$,$s_{2}$,...,$s_{n}$}. $T_{0}$ is the target set deployed, and *m* is the number of targets in $T_{0}$={$t_{1}$,$t_{2}$,...,$t_{m}$}. *B* is the set of sinks, and *t* is the number of sinks, *B*={$b_{1}$,$b_{2}$,...,$b_{t}$}. *k* is the required coverage degree. E${}_{remain}$ is the remaining energy of the sensor node. E${}_{Init}$ is the initial energy of the sensor node. E${}_{minal}$ is the smallest energy that can be used in the current connected cover set that is being built. C${}_{cur}$ is the currently-connected cover set. $T_{S_{i}}$ is the set of targets covered by the sensor node $S_{i}$. $T_{S_{i}}^{u}$ is the set of targets in $T_{S_{i}}$ that cannot be covered by sensors in $C_{cur}$. $T_{S_{i}}^{k}$ is the set of targets in $T_{S_{i}}$ that can be *k*-covered by sensors in $C_{cur}$. $T_{S_{i}}^{T_{0}}$ denotes the targets in $T_{0}$ that a sensor $S_{i}$ covers. $T_{S_{i}}^{T_{cur}}$ denotes the targets in $T_{cur}$ that a sensor $S_{i}$ covers. RID is the ID set of relay nodes. $Flag_{S_{i}}$ is to ensure connectivity between the sensor node $S_{i}$ and the sink $b_{j}$. When $Flag_{S_{i}}=false$, $S_{i}$ is not connected to all of the sinks in B. Otherwise, $S_{i}$ is connected to a sink by either one hop or multi-hops.

Symbols and parameters used in the centralized connected target *k*-coverage algorithm (CCTC${}_{k}$) and the distributed connected target *k*-coverage algorithm (DCTC${}_{k}$) are listed in the Table 1.

**Table 1:** Symbols and parameters.

Symbols	Description
*E*${}_{remain}$	The remaining energy of sensor node
*E*${}_{Init}$	The initial energy of sensor node
*E*${}_{minal}$	The smallest energy can use in $C_{cur}$ being built
*C*${}_{cur}$	The currently connected cover set
$T_{S_{i}}$	The set of targets covered by the sensor node $S_{i}$
$T_{S_{i}}^{u}$	Targets cannot be covered by sensors in $C_{cur}$
$T_{S_{i}}^{T_{0}}$	The targets in $T_{0}$ that $S_{i}$ covers
$T_{S_{i}}^{T_{cur}}$	The targets in $T_{cur}$ that $S_{i}$ covers
$T_{S_{i}}^{k}$	$S_{i}$ can *k*-cover targets
*RID*	The ID set of relay nodes
*Flag*	Boolean variable

Our proposed algorithm selects an active sensor node for a connected cover set based on the following three aspects: (i) the algorithm needs to promote the candidates that can cover as few of the already *k*-covered targets as possible; that is to select the sensor node that can cover as many targets only covered by itself as possible; (ii) the algorithm needs to favor the candidates that have more battery life available; and (iii) the algorithm needs to promote candidates that cover a target that is not *k*-covered by sensor nodes in the existing selected set. Based on these three aspects, we define our $SET(S_{i})$ function as follows:

(1) $$SET\left(S_{i}\right)=1-\frac{\left|coverstate(S_{i})\right|}{|T_{cur}|}\times a-\frac{E_{remain}}{E_{Init}}\times b-\frac{|T_{s_{i}}^{u}|}{|T_{s_{i}}|}\times c$$

where $coverstate(S_{i})$ describes the coverage state of a sensor, *i.e.*, it measures the number of covered targets and *k*-uncovered in relation to the number of already *k*-covered targets the sensor monitors. It is computed by the following formula,

(2) $$coverstate\left(S_{i}\right)=\frac{T_{S_{i}}^{uk}}{{\left(T_{S_{i}}^{T_{o}}-T_{S_{i}}^{T_{cur}}+1\right)}^{\lambda }}$$

where $T_{S_{i}}^{uk}$ is the number of targets covered by the sensor that have not been *k*-covered previously, $T_{S_{i}}^{T_{0}}-T_{S_{i}}^{T_{cur}}$ is the number of already *k*-covered targets that the sensor is capable of covering, and $\lambda =\frac{T_{0}-T_{cur}}{T_{0}}$. The objectives of the $coverstate(S_{i})$ function are to promote nodes that cover as many uncovered targets as possible, and to penalize nodes that cover already *k*-covered targets. Clearly, the two objective are satisfied from the fraction $\frac{T_{S_{i}}^{uk}}{T_{S_{i}}^{T_{o}}-T_{S_{i}}^{T_{cur}}+1}$. We add one to the denominator to avoid division by zero. The use of $\lambda =\frac{T_{0}-T_{cur}}{T_{0}}$ (λ∈[0,1)) gradually increases the penalty on nodes that cover already *k*-covered targets.

In the $SET(S_{i})$ function, the parameters *a*, *b* and *c* are constants, with a,b,c∈(0,1) and a+b+c=1. Their values can be tuned according to the nature of the examined problem. For example, by increasing *a*, the algorithm pays more attention to the coverage status of sensors and produces cover sets with a smaller number of nodes. A larger *b* value would prioritize the selection of nodes with a higher remaining energy, while a larger *c* would favor the nodes that cover more targets with smaller coverage degrees currently.

### 4.1. Centralized Connected Target *k*-Coverage Algorithm

The key characteristic of the proposed algorithm is a uniform node selection strategy that tries to select the smallest $SET(S_{i})$ sensor. We first construct a cover set that can *k*-cover all targets. After that, we determine whether each sensor in the cover set can communicate with the sink nodes by one-hop or multi-hop neighbors. If a sensor cannot directly communicate with the sink, it needs to find an appropriate neighbor as its relay node. Similarly, a relay node may also need to find its relay node to continue relaying the sensed data to the sink. A connected cover set is formed until it is not necessary to include more sensors to ensure that the network is connected. If the remaining energy of a sensor is less than $E_{minal}$, that is it cannot do sensing work and relaying work, it goes into the sleep state. The following pseudo-code gives the details of our proposed centralized connected target *k*-coverage Algorithm 1 -CCTC${}_{k}$.

**Algorithm 1.** Centralized connected target *k*-coverage algorithm (CCTC${}_{k}$)          **begin**          C=ϕ          $S_{avail}=S$          **for** (S${}_{i}\in S_{avail}$) **do**               $E_{remain}=E_{Init}$               **if**
$S_{i}$ is connected with sink node **do**                   $Flag_{S_{i}}$=true               **else**                   $Flag_{S_{i}}$=false               **end**          **end**          **while** ($S_{avail}\neq \phi$) **do**               $S_{cur}=S_{avail}$               $T_{cur}=T_{0}$               $C_{cur}=\phi$               **Coverage:**               **while** ($T_{cur}\neq \phi$) **do**                   $S_{selected}=\phi$                   $SET_{min}=c$         c is a large constant                   **for** (S${}_{i}\in S_{cur}$) **do**                        Calculate $SET(S_{i})$                        **if**
$SET_{min}>SET\left(S_{i}\right)$
**do**                           $SET_{min}=SET\left(S_{i}\right)$                           $S_{selected}=S_{i}$                        **end**                   **end**                   **if**
$S_{selected}=\phi$
**do**                        $S_{avail}=\phi$                        $T_{cur}=\phi$                   **end**                   **Calculates**
$T_{S_{selected}}^{k}$                   $T_{cur}=T_{cur}-T_{S_{selected}}^{k}$                   $S_{cur}=S_{cur}-S_{selected}$                   $E_{remain}=E_{remain}-E_{selected}$                   **if**
$E_{remain}<E_{minal}$
**do**                        $S_{avail}=S_{avail}-S_{selected}$                   **end**                   $C_{cur}=C_{cur}\cup S_{selected}$              **end**              **Connectivity:**              $C_{temp}=C_{cur}$              **for** (S${}_{i}\in C_{temp}$) **do**                   **while** ($Flag_{S_{i}}$=false) **do**                      $S_{i}$ selected an appropriate neighbor $S_{j}$ to relay the sensed data                        $C_{cur}=C_{cur}\cup S_{j}$                        **if** ($Flag_{S_{j}}$=false) **do**                            $S_{i}=S_{j}$                        **end**                   **end**              **end**          **end**

### 4.2. A Distributed Connected Target *k*-Coverage Algorithm

**Figure 6:**
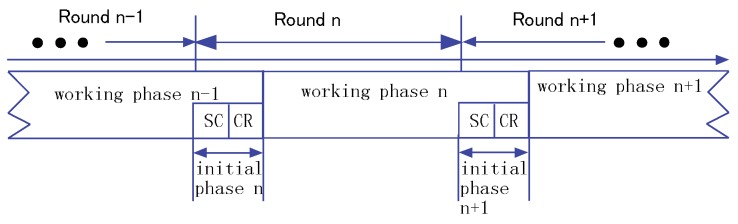
The lifetime of the network.

We divide the lifetime of an HWSN into rounds. As shown in Figure 6, time is divided into rounds of equal length. A round consists of an initial phase and a working phase. The initial phase is further divided into a sensing coverage (SC) subphase and a communication relay (CR) subphase. Let D${}_{Init}$, D${}_{SC}$ and D${}_{CR}$ denote the durations of the initial phase, the SC subphase and the CR subphase, respectively. Clearly, D${}_{Init}$ = D${}_{SC}$ + D${}_{CR}$. Note that D${}_{Init}$ is much shorter than the duration of a round.

During D${}_{SC}$, each sensor determines whether it should be in active state or wait state. During D${}_{CR}$, if one sensor node becomes active and cannot communicate with the sink, it still needs to find an appropriate neighbor to relay the sensed data. Similarly, the relay node needs to find its relay node to continue relaying the sensed data to the sink. In D${}_{SC}$, sensor nodes are activated to cover all of the targets. D${}_{CR}$ is only used for choosing relay nodes, so as to guarantee network connectivity. The working phase begins at the end of the initial phase and ends before the initial phase of the next round, such that the targets can be continuously monitored. In the working phase, the sensor nodes in the active state begin sensing work, and the nodes in the relaying state prepare to relay sensed data.

The messages used during the execution of DCTC${}_{k}$ are given as follows.

- *Active* is the message sent when one sensor node activates itself as one member of a cover set. The message includes the ID of the sender, the cover set $C_{cur}$ currently produced and the target set $T_{S_{i}}$ covered by the sender.
- *Candidate* is the message sent when one sensor node $S_{i}$ receives an *Ask* message and notices that the value of Flag is true. The *Candidate* message is important for the connectivity of the network. The message includes the ID of the sender, its state, and its energy.
- *Connect* is the message sent by a sensor node after receiving the *Candidate* messages. The sensor node selects the best candidate node and sends a *Connect* message to the candidate. The message includes the ID of the candidate node and its state. Nodes who send *Connect* messages set their Flags to true.
- *Sink* is the message sent by a sensor node to check its connectivity to the sinks. Each sensor node sends a *Sink* message to sinks to detect whether it is connected to the sinks.
- *Reply* is the message sent by sinks. Sinks broadcast *Reply* messages to sensor nodes. Sensor nodes that receive the message are connected to the sink, and their Flags are set to true.
- *Ask* is the message sent by the nodes that are not connected to a sink. The message includes the ID of the sender.

**Figure 7:**
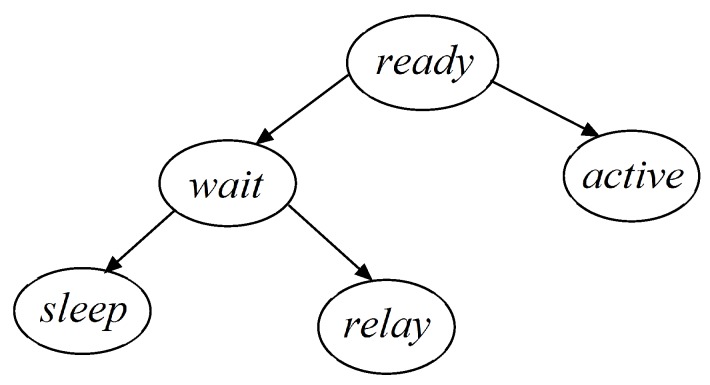
The state transition diagram.

As we have mentioned in the previous section, each sensor node can be in one the five state: *ready*, *wait*, *sleep*, *relay* and *active*. In DCTC${}_{k}$, each sensor node $S_{i}$ starts with the *ready* state. $S_{i}$ executes our proposed algorithm to determine whether it goes into the *active* state or the *wait* state. If $S_{i}$ goes into the *wait* state and receives a *Connect* message, it goes into the *relay* state. Otherwise, it goes into the *sleep* state. The state transition diagram is given in Figure 7.

In each round, every sensor node in the *ready* state executes the algorithm to determine whether it goes into the *sleep* state, *active* state or *relay* state. The main idea of the algorithm is to set a waiting decision time ($W_{t}$) for each sensor node. The node that has more energy and covers more targets has a smaller waiting decision time and, thus, has the priority to go into *active* state. If a sensor’s energy is less than $E_{minal}$, it goes into *sleep* state.

In the coverage portion of the algorithm, a sensor node decides whether it goes into the *wait* state or the *active* state. Initially, each node $S_{i}$ is in the ready state. A node first calculates the targets it covers ($T_{S_{i}}$) and its waiting decision time $W_{t}$. Update $T_{S_{i}}$ and $W_{t}$ dynamically. If the targets covered by $S_{i}$ have been *k*-covered by other nodes, $T_{S_{i}}$ is empty, and $S_{i}$ goes into the *wait* state. Otherwise, $S_{i}$ goes into the *active* state, and $S_{i}$ broadcasts an *Active* message to its neighbors. If a sensor node in the *wait* state receives a *Connect* message from its neighbors, it goes into the *relay* state. Otherwise, it goes into the *sleep* state. If a sensor node in the *active* state receives a *Connect* message from its neighbors, it remains in the *active* state.

Then, the algorithm selects relay nodes to guarantee the connectivity of the network in the connectivity portion. To be specific, every sensor node that is in the ready state and whose Flag is false broadcasts a *Sink* message. The nodes who receive a *Reply* message set their Flag values to true to indicate that they can communicate with sinks. If a sensor node’s $Flag_{S_{i}}=false$, $S_{i}$ needs to find a relay node. Thus, $S_{i}$ sends an *Ask* message to its neighbors. If a node’s $Flag_{S_{i}}=true$ and it receives the *Ask* message, it sends a *Candidate* message to the sender of the *Ask* message. If a sensor node receives *Candidate* messages, it selects a candidate node and sends a *Connect* message to the candidate. If all candidate nodes are in the *wait* state, it selects the one with the most energy. The pseudo-codes of DCTC${}_{k}$ are listed in Algorithm 2.

**Algorithm 2.** Distributed connected target *k*-coverage algorithm (DCTC${}_{k}$))    **begin**     Flag←false     $S_{i}$ sends Sink message     **if**
$E_{remain}<E_{minal}$
**do**     $S_{i}$ enters into sleep state     **end****Coverage:**         Calculate $T_{S_{i}}$         Calculate waiting decision time $W_{t}=T\times \left(1-\frac{\left|coverstate(S_{i})\right|}{|T_{cur}|}\times a-\frac{E_{remain}}{E_{Init}}\times b-\frac{|T_{s_{i}}^{u}|}{|T_{s_{i}}|}\times c\right)$         $C_{cur}=\phi$         **while**
$W_{t}$ has not expired **do**              **if**
$S_{i}$ receives an Active message from a neighbor $S_{j}$
**do**                  $C_{cur}=C_{cur}\cup S_{j}$                  **for**
$t_{i}\in T_{S_{i}}$
**do**                      **if**
$t_{i}$ is uncovered by sensors in $C_{cur}$
**do**                      $T_{S_{i}}^{u}=T_{S_{i}}^{u}+t_{i}$                      **end**                      **if**
$t_{i}$ is *k*-covered by sensors in $C_{cur}$
**do**                       $T_{S_{i}}=T_{S_{i}}-t_{i}$                      **end**                 **end**                 **if**
$T_{S_{i}}=\phi$
**do**                      $S_{i}$ enters wait state                 **else**                      $W_{t}=T\times \left(1-\frac{\left|coverstate(S_{i})\right|}{|T_{cur}|}\times a-\frac{E_{remain}}{E_{Init}}\times b-\frac{|T_{s_{i}}^{u}|}{|T_{s_{i}}|}\times c\right)$                 **end**             **end**        **end**         **if**
$S_{i}$ receives a Relay message from the sink **do**             $Flag_{S_{i}}=true$         **end**         **if**
$S_{i}$ in ready state **do**             $S_{i}$ broadcasts an Active message to its neighbors             $S_{i}$ changes into active state         **end****Connectivity:**         **while**
$Flag_{S_{i}}=false$
**do**             $S_{i}$ broadcasts an ASK message to its neighbors             **if**
$S_{i}$ receives an ASK message from $S_{j}$
**do**                 **if**
$Flag_{S_{i}}=true$
**do**                 $S_{i}$ sends a Candidate message to $S_{j}$                 **end**             **end**             **if**
$S_{i}$ receives a Candidate message from $S_{j}$
**do**                 $TID=TID\cup S_{j}$             **end**             Add $S_{j}$ to RID, the remaining energy if which is larger than others in TID             $Flag_{S_{i}}=true$             $S_{i}$ broadcasts a Connect message             **if**
$S_{i}$ receives a Connect message from $S_{j}$
**do**                 **if**
$S_{i}\in RID$
**do**                     $S_{i}$ broadcasts a Active message                 **end**             **end**         **end**

## 5. Algorithm Analysis

In the CCTC${}_{k}$ algorithm, we need to calculate $SET(S_{i})$ so as to select sensors. Each time, the smallest $SET(S_{i})$ of sensor $S_{i}$ is added to $C_{cur}$. Next, $S_{i}$ can be removed from $S_{cur}$, and targets *k*-covered can be removed from $T_{cur}$. After forming $C_{cur}$, we determine whether a sensor in the $C_{cur}$ can communicate with the sink. If a sensor cannot communicate with the sink, it needs to find an appropriate neighbor to relay the sensed data. Similarly, the relay node that cannot directly communicate with the sink also needs to find its relay node to the sink. Hence, the network is connected. If there are still sensors available to utilize, an empty cover set ($C_{cur}$) is created and initialized to the set of currently uncovered targets $T_{cur}$ (with the targets found in $T_{0}$) and the set of currently available sensors $S_{cur}$ (with the sensors found in $S_{avail}$). The process is repeated until there are no sensors available to utilize.

**Theorem 1.**  *The centralized connected target k-coverage algorithm is capable of generating at least one connected cover set, if one exists*.

**Proof.**  The proofs are divided into two parts. Firstly, we prove that the active sensor nodes generated in the coverage part of the CCTC${}_{k}$ can *k*-cover all the targets. Assume that there is a target $t_{i}$ that is *m*-covered by active sensor nodes and m<k. According to the assumptions made in Section 3, all of the targets can be *k*-covered by the sensor nodes in *S*. Thus, there exist at least *k* sensor nodes that can *k*-cover each target in *T*. Suppose that $t_{i}$ is covered by y+1 sensor nodes, denoted by $S_{i}$, $S_{i_{1}}$,...,$S_{i_{y}}$, where y≥k-1. $S_{i_{2}}$,...,$S_{i_{m}}$ are the nodes in the *active* state. Additionally, other k-m sensor nodes are not in the *active* state. Let $SET(S_{i})$ be the smallest sensor among the y+1 sensors. $S_{i}$ is the member of the covet set and goes into active state according to CCTC${}_{k}$. The process of $S_{i}$ is applied to other sensor nodes, $S_{i_{m+1}}$,...,$S_{i_{k}}$. Hence, $t_{i}$ is *k*-covered, which contradicts the hypothesis. Thus, the cover set produced by the algorithm can *k*-cover all of the targets in *T*.

By contradiction, we prove that the cover set produced by the algorithm can communicate with the sink. Initially, the value of *Flag* of each sensor node in *S* is false. These sensor nodes are directly communicating with the sink. The value of Flag of these sensors is true; otherwise, the value of Flag of other sensors is false. Assume there is an active sensor node that is not connected with the sink, denoted by $S_{i}$. According to the algorithm, $S_{i}$ needs to find an appropriate neighbor as its relay node. The algorithm loops until the Flag of $S_{j}$ is true, if the Flag of $S_{j}$ is still false. According to the assumption in Section 3, all of the sensors are connected with the sink by one-hop or multi-hops. Thus, the Flag of $S_{j}$ will be true after several loops. $S_{j}$ is added to the cover set, if there exists one neighbor node whose Flag is true. Then, $S_{i}$ is connected with a sink via multi-hops, which is against the hypothesis. Thus, all of the sensor nodes in the connected cover set generated by CCTC${}_{k}$ are connected with the sink. ☐

DCTC${}_{k}$ produces connected cover sets by considering coverage and connectivity separately, including a *Coverage* algorithm and a *Connectivity* algorithm. Firstly, in the *Coverage* algorithm, the nodes in the *active* state can guarantee *k*-coverage of all of the targets. However, these active sensor nodes cannot guarantee the connectivity of the network. Thus, in the *Connectivity* algorithm, the isolated nodes are connected with the sinks via some relay nodes. Each sensor node can obtain its state according to our algorithm. The sensor nodes that are in the *active* state or *relay* state form a connected cover set. In Theorem 2, we prove that the active sensor nodes generated in the coverage part of the algorithm can *k*-cover all of the targets.

**Theorem 2.**  *The cover set C returned by the Coverage algorithm can k-cover all of the targets in T*.

**Proof.**  We firstly prove that the active sensor nodes produced in the *Coverage* algorithm of DCTC${}_{k}$ can *k*-cover all of the targets in *T*. By contradiction, assume that there is a target $t_{i}$ that is *m*-covered by active sensor nodes and m<k. According to the assumption in Section 3, all of the targets can be *k*-covered by the sensor nodes in *S*, and the network constructed is connected. Thus, there exist at least *k* sensor nodes that can *k*-cover each target in *T*. Suppose that $t_{i}$ is covered by x+1 sensor nodes, denoted by $S_{i}$, $S_{i_{1}}$,..., $S_{i_{x}}$, where x≥k-1, and these sensor nodes are connected. Assuming that the target sets containing $t_{i}$ are denoted by $T_{s_{i}}$,$T_{s_{i_{1}}}$,...,$T_{s_{i_{x}}}$ and $S_{i_{1}}$, $S_{i_{2}}$,...,$S_{i_{m}}$ are the nodes in the *active* state. Additionally, other k-m sensor nodes are not in the *active* state. Let $W_{t}$ be the smallest sensor among the x+1 sensors. According to the Coveragepart of the algorithm, $t_{i}$ is still restored in $T_{s_{i}}$ since $t_{i}$ is not *k*-covered. $S_{i}$ goes into the *active* state. The process of $S_{i}$ goes the same with other sensor nodes, $S_{i_{m+1}}$,...,$S_{i_{k}}$. Then, $t_{i}$ is *k*-covered, which contradicts the hypothesis. Thus, the cover produced in the Coverage algorithm can *k*-cover all of the targets in *T*. ☐

The algorithm can guarantee not only the *k*-coverage of all targets, but also the connectivity of the network.

**Theorem 3.**  *All of the sensor nodes in the connected cover set generated by DCTC${}_{k}$ are connected with the sink*.

**Proof.**  By contradiction, assume there is an active sensor node that is not connected with the sink, denoted by $S_{i}$. Initially, the value of Flag of each sensor node in *S* is false. According to the algorithm, the sink broadcasts a *Reply* message. The value of the Flag of sensor nodes that receive the *Reply* message is set to true. These sensor nodes are directly connected with the sink. The values of Flag of other sensor nodes, including $S_{i}$, are false. After the coverage part in the algorithm, the sensor nodes are in the *active* state or in the *wait* state. Thus, $S_{i}$ broadcasts an *Ask* message to its neighbors. If there exists no sensor nodes that receive such a message and its Flag is true at the moment; the Flag of $S_{i}$ is still false; and the *Connectivity* algorithm loops until the Flag of $S_{i}$ is true. According to the assumption in Section 3, all of the sensor nodes are connected with the sinks via one-hop or multi-hops. Thus, the Flag of $S_{i}$ will be true after several loops. If there exist some neighbor nodes whose Flag are true, they send a *Candidate* message to $S_{i}$. Then, $S_{i}$ selects one candidate node $S_{j}$, sends a *Connect* message to $S_{j}$ and sets its $Flag_{i}=true$. $S_{j}$ is also selected in the connected cover set. Then, $S_{i}$ is connected with a sink via multi-hops, which is against the hypothesis. Thus, all of the sensor nodes in the connected cover set generated by DCTC${}_{k}$ are connected with the sink. ☐

As DCTC${}_{k}$ is a distributed algorithm, the global information cannot be obtained. There will be more redundant sensor nodes in the connected cover sets produced by DCTC${}_{k}$.

**Theorem 4.**  *The time complexity of DCTC${}_{k}$ is O(pq), and the message complexity of DCTC${}_{k}$ is O(5n), where p is the maximum number of targets covered by a sensor node, q is the maximum number of communication neighbor nodes of a sensor node and n is the number of sensor nodes deployed*.

**Proof.**  At the beginning of the *Coverage* algorithm of DCTC${}_{k}$, each sensor node $S_{i}$ needs to calculate the target set $T_{s_{i}}$. According to the *Coverage* part of DCTC${}_{k}$, the time complexity of the determination of whether the targets are covered by other nodes is O(p). Thus, the time complexity of the Coverage algorithm is O(pq). While the time complexity of the *Connectivity* part is O(q). Thus, the time complexity of DCTC${}_{k}$ is O(pq). In each round, when a sensor node activates itself, it broadcasts an *Active* message. Then, the node goes into the *active* state. Assume all of the sensor nodes go into the *active* state; each node will broadcast an *Active* message. Then, the maximum communication complexity of DCTC${}_{k}$ in broadcasting active coverage is O(n). Similarly, the communication overhead of exchanging *Connect*, *Ask*, *Sink* and *Candidate* messages in the Connectivity algorithm is also O(n). Therefore, the maximum communication overhead of DCTC${}_{k}$ is O(5n). ☐

According to Theorem 4, the computational complexity of the coverage portion of the algorithm is only influenced by the density of the deployed sensor nodes and targets. In general, with the redundancy of deployment, a small subset of sensor nodes broadcasts messages; the communication overhead is far less than O(n).

## 6. Evaluation

In this section, we evaluate the performance of our proposed algorithms via simulation. We simulate a stationary network with sensor nodes and targets deployed randomly in a 400 m × 400 m region. Sinks are uniformly deployed in the sensing region. Additionally, we consider the following parameters.

1. The initial battery energy of each node is between 70 J and 90 J. The power used for sensing is 64 mW [33]. The power used for sensing, computing, controlling and processing is 383 mW. The power used for transmitting data, receiving data, the wait state and the sleeping state is 700 mW, 370 mW, 340 mW and 40 mW, respectively.
2. The sensing radii are between 50 m and 60 m, and the communication radii are between 100 m and 120 m. The parameters *a*, *b* and *c* are 0.4, 0.3 and 0.3, respectively.
3. We divide the lifetime of the network into rounds. In each round, there is the initial phase and the working phase.

**Figure 8:**
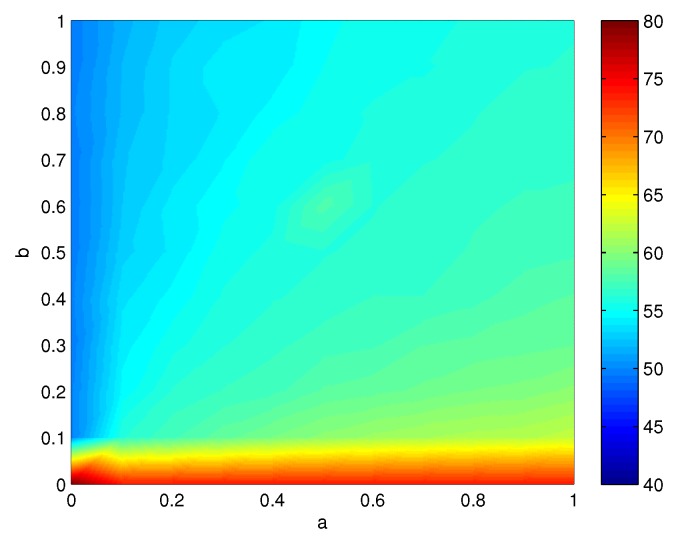
The relation between *a*, *b* and the number of generated connected cover sets.

The parameters *a*, *b* and *c* are constants, satisfying a,b,c∈(0,1) and a+b+c=1. Their values can be tuned according to the request of the examined problem. For example, by increasing *a*, the algorithm pays more attention to the coverage status of sensors and produces connected cover sets with a smaller number of nodes. A larger *b* would prioritize the selection of nodes with a higher remaining energy, while a larger *c* would select the nodes that cover more targets with smaller coverage degrees currently. Figure 8 shows the relation between *a*, *b* and the number of generated connected cover sets. The *c* is equal to 1-a-b. Here, there are 300 sensor nodes and 40 targets randomly deployed in the sensing region. We can see that when *a* and *c* become large, the number of connected cover sets generated also grows large.

**Figure 9:**
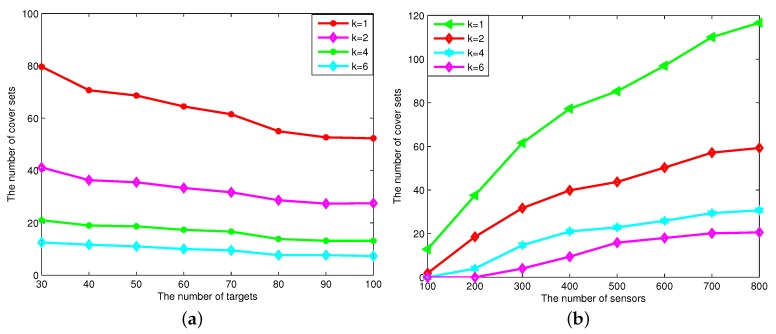
The number of connected cover sets generated *vs.* the number of targets and the number of sensor nodes. (**a**) The number of cover sets changes when the number of targets changes; (**b**) the number of cover sets changes when the number of sensors changes.

Figure 9 shows the number of connected cover sets generated under various numbers of targets and different numbers of sensor nodes for the DCTD${}_{k}$. We can see that the number of connected cover sets decreases as the coverage degrees increase, since it needs more sensor nodes to multi-cover all of these targets. When the number of targets increases, the number of connected cover sets decreases. The reason is that the number of sensor nodes for *k*-covering all of these targets becomes greater as the number of targets increases. When the number of sensor nodes increases, the number of connected cover sets increases. The reason is that the number of sensor nodes generating connected cover sets for *k*-covering these targets increases as the number of sensor nodes increases.

**Figure 10:**
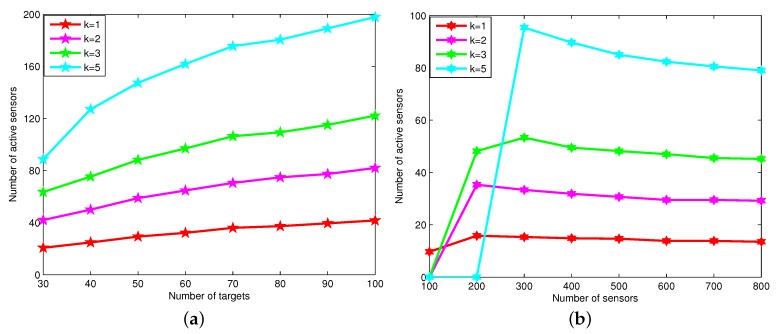
The average number of active nodes *vs.* the number of targets and sensor nodes. (**a**) The average number of active nodes changes when the number of targets changes; (**b**) the average number of active nodes changes when the number of sensor nodes changes.

Figure 10 shows the average number of active nodes under various different numbers of targets and sensor nodes for the DCTD${}_{k}$. We can see that the number of sensor nodes increases as the coverage degrees increase, since it needs more sensor nodes to multi-cover all of these targets. Obviously, when the number of targets increases, the number of sensor nodes increases. When the number of sensor nodes increases, the number of active sensor nodes decreases. The reason is that when the number of sensor nodes is large, the network is dense enough to choose optimal sensor nodes for *k*-covering the targets.

**Figure 11:**
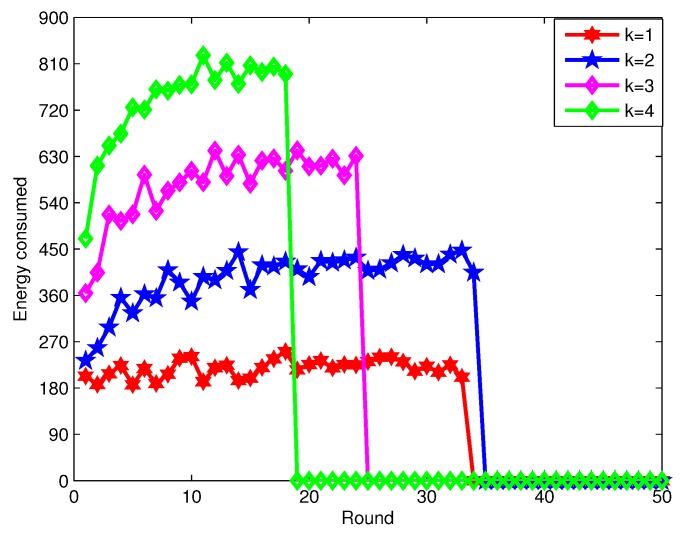
The energy consumed by all of the sensor nodes by the connected cover set in every round.

Figure 11 shows the energy consumed in every round by the connected cover set. Here, the number of sensor nodes is 300, and the number of targets is 50. The sensing radii of sensor nodes are between 40 m and 45 m, and the communication radii are between 80 m and 90 m. The consumed energy increases when the coverage degrees required increase, since it needs more sensor nodes to *k*-cover these targets and it takes more energy for sensing and communication tasks.

Next, we compare our algorithm DCTC${}_{k}$ to EETC [23] and LADCS [28]. Here, the sensing radii of sensor nodes are between 40 m and 45 m, and the communication radii are between 80 m and 90 m. The coverage degree is one.

**Figure 12:**
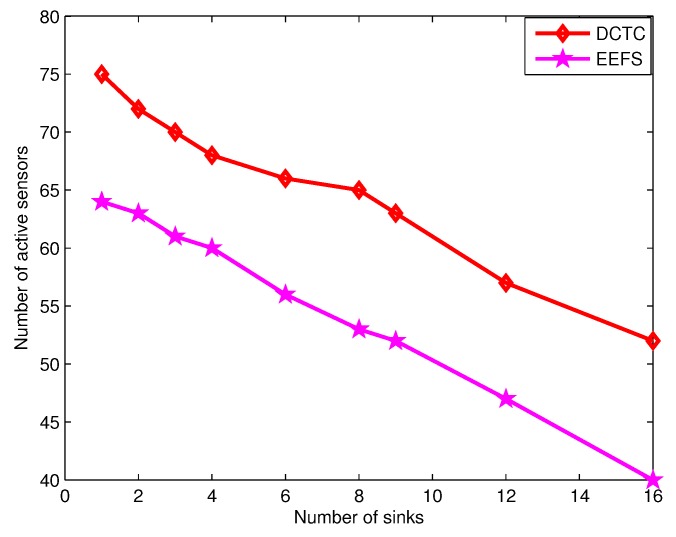
The number of connected cover sets *vs.* the number of sinks.

In Figure 12, we can see the number of connected cover sets generated under various numbers of sinks. We can see that when the number of sinks increases, the number of connected cover sets increases, as there are fewer sensor nodes selected as relay nodes. In addition, our algorithm can generate more connected cover sets than that of EETC. The reason is that our algorithm considers the comprehensive performance of sensor nodes, including sensing state, remaining energy and the coverage of targets covered by them. However, EETC does not consider the coverage of targets covered by sensor nodes when selecting a sensor node. Moreover, our algorithm DCTC${}_{k}$ generates less relay nodes than EETC in the connectivity portion of the algorithm.

**Figure 13:**
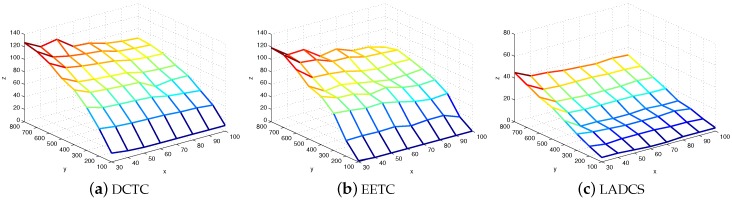
The number of connected cover sets generated in DCTC, EETCand LADCS.

Figure 13 shows the number of connected cover sets under different numbers of targets and sensor nodes. The **x** axis represents the number of targets; the **y** axis is the number of sensor nodes; and the **z** axis is the number of connected cover sets generated. We can infer that our algorithm can generate more connected cover sets compared to EETC and LADCS. That is because our algorithm emphasizes the balance of the coverage performance of sensor nodes (such as the remaining power and covered targets). However, the EEFC scheme just considers the remaining energy. The LADCS scheme produces a disjoint covet set.

**Figure 14:**
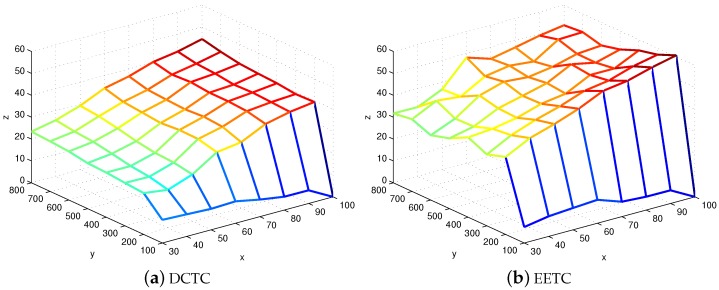
The average number of active sensor nodes in connected cover sets.

Figure 14 shows the average number of active sensor nodes in connected cover sets when the coverage degree is one. The **x** axis represents the number of targets deployed; the **y** axis is the number of sensor nodes deployed; and the **z** axis is the average number of active sensor nodes. From the figure, we can conclude that our algorithm requires less active sensor nodes in connected cover sets to cover targets compared to EETC. The reason is that the EETC scheme just considers the remaining energy, while the DCTC scheme considers the sensing capabilities of the sensor node, its remaining energy and the case of *k*-coverage of the targets covered by it.

**Figure 15:**
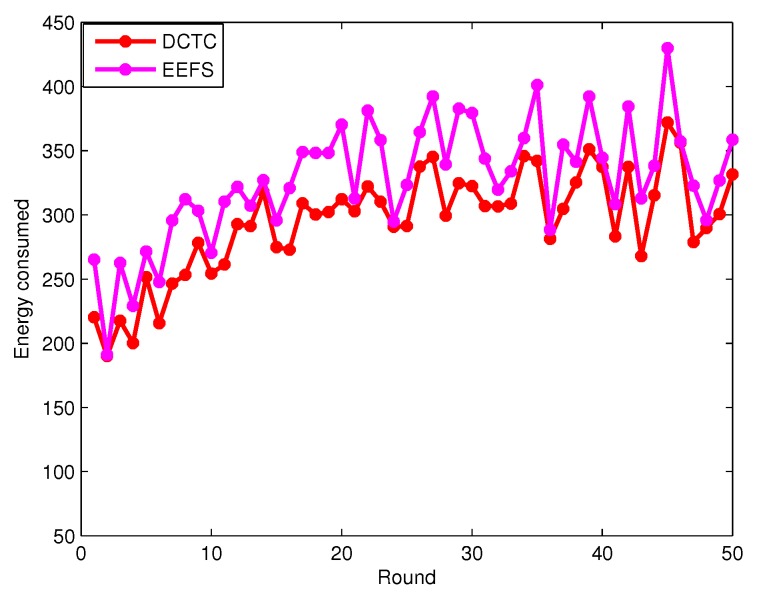
The energy consumed by all of the sensor nodes by the connected cover set in each round.

Figure 15 shows the energy consumed by sensor nodes in the connected cover set in each round when the coverage degree is one. The DCTC${}_{k}$ algorithm can achieve the load balance of sensor nodes and prolong the network lifetime by considering the sensing capabilities, remaining battery life and the *k*-coverage targets of each sensor node, which need to select less sensor nodes in each connected cover set. However, EEFS just considers the sensor’s remaining energy, which needs to select more sensors in each connected cover set. Thus, We can conclude that our algorithm DCTC${}_{k}$ consumes less energy compared to EETC in each round.

## 7. Conclusions

In the paper, we propose a centralized connected target *k*-coverage algorithm (CCTC${}_{k}$) and a distributed connected target *k*-coverage algorithm (DCTC${}_{k}$) for energy-efficient connectivity and coverage maintenance in HWSNs. These two proposed algorithms aim at producing sufficient connected cover sets, such that all of the targets in the target set *T* are covered by at least *k* active sensor nodes. At the same time, our algorithms aim to minimize the necessary total number of active sensor nodes in the connected cover set and guarantee that each sensor node in the connected cover set can be connected to the sinks. The sensor selection strategy is based on a sensor’s sensing capabilities, remaining battery life and the percentage of targets covered by it. Since our proposed algorithms consider *k*-coverage and one-connectivity at the current stage, we plan to study multi-connectivity in the future to enhance the robustness of networks.
